# Relationship between Food Insecurity and Mortality among HIV-Positive Injection Drug Users Receiving Antiretroviral Therapy in British Columbia, Canada

**DOI:** 10.1371/journal.pone.0061277

**Published:** 2013-05-27

**Authors:** Aranka Anema, Keith Chan, Yalin Chen, Sheri Weiser, Julio S. G. Montaner, Robert S. Hogg

**Affiliations:** 1 British Columbia Centre for Excellence in HIV/AIDS, St. Paul's Hospital, Vancouver, British Columbia, Canada; 2 Department of Medicine, Faculty of Medicine, University of British Columbia, Vancouver, British Columbia, Canada; 3 Division of HIV/AIDS, San Francisco General Hospital, University of California San Francisco, San Francisco, California, United States of America; 4 Center for AIDS Prevention Studies, University of California San Francisco, San Francisco, California, United States of America; 5 Faculty of Health Sciences, University of British Columbia, Burnaby, British Columbia, Canada; CUNY, United States of America

## Abstract

**Objectives:**

Little is known about the potential impact of food insecurity on mortality among people living with HIV/AIDS. We examined the potential relationship between food insecurity and all-cause mortality among HIV-positive injection drug users (IDU) initiating antiretroviral therapy (ART) across British Columbia (BC).

**Methods:**

Cross-sectional measurement of food security status was taken at participant ART initiation. Participants were prospectively followed from June 1998 to September 2011 within the fully subsidized ART program. Cox proportional hazard models were used to ascertain the association between food insecurity and mortality, controlling for potential confounders.

**Results:**

Among 254 IDU, 181 (71.3%) were food insecure and 108 (42.5%) were hungry. After 13.3 years of median follow-up, 105 (41.3%) participants died. In multivariate analyses, food insecurity remained significantly associated with mortality (adjusted hazard ratio [AHR] = 1.95, 95% CI: 1.07–3.53), after adjusting for potential confounders.

**Conclusions:**

HIV-positive IDU reporting food insecurity were almost twice as likely to die, compared to food secure IDU. Further research is required to understand how and why food insecurity is associated with excess mortality in this population. Public health organizations should evaluate the possible role of food supplementation and socio-structural supports for IDU within harm reduction and HIV treatment programs.

## Introduction

Despite the tremendous benefits of antiretroviral therapy (ART) use on HIV disease progression and survival [1], [2], micro- and macronutrient malnutrition remain strong independent predictors of mortality among HIV-positive individuals in both high and low resource settings [3], [4], [5], [6], [7], [8], [9], [10], [11], [12], [13]. A growing body of evidence suggests that socio-economic determinants may also adversely impact survival among people living with HIV/AIDS [14], [15]. More recently, our study team found that among HIV-positive individuals receiving ART in Canada, being food insecure and underweight was independently associated with a 1.94 increased risk of non-accidental death, compared to being food secure and of normal weight [16]. Poor dietary diversity, a component of food insecurity, has been associated with mortality among ART-naïve individuals in Uganda [17]. These studies suggest that food insecurity warrants prioritization by public health programs and policies for HIV-infected populations.

Illicit drug use is a well-known risk factor for food insecurity and poor nutritional status. Drug addiction alters dietary consumption patterns, leading individuals to eat fewer meals [18], to often skip meals for an entire day [19], and to rely on food distribution services for subsistence [20]. Studies have found that the diets of illicit drug users tend to be calorically insufficient and poor in quality [18], resulting in diverse micro- and macronutrient deficiencies [19], [21], [22], [23], [24]. Food insecurity is theorized to be linked to adverse HIV outcomes through distinct nutritional, mental health and behavioral pathways [25]. Studies in urban HIV-positive populations receiving HIV treatment, including a high proportion of illicit drug users, have found that food insecurity is associated with HIV-related wasting [19], virologic non-suppression [26], [27] and poor immunologic response to ART [27], [28], [29].

To our knowledge, no studies have examined whether food insecurity increases risk of excess death in this population. This study therefore aimed to assess the potential relationship between food insecurity and all-cause mortality among HIV-positive injection drug users (IDU) initiating ART across BC. In light of previous findings regarding the relationship between food insecurity and mortality in the general HIV-population in this setting [16], and theorized nutritional mechanisms linking these [25], this study hypothesized that food insecurity is independently associated with all-cause mortality.

## Methods

### Study Sample: HIV/AIDS Drug Treatment Program (DTP)

Survey data for this analysis were obtained from the provincial HIV/AIDS Drug Treatment Program (DTP) administrative database. In BC, antiretrovirals have been distributed free of charge to HIV-positive individuals since 1986, and coordinated centrally since 1992 by the British Columbia Centre for Excellence in HIV/AIDS (BC-CfE) HIV/AIDS DTP, located at St. Paul's Hospital in Vancouver. Details of the HIV/AIDS DTP have been described elsewhere [1]. In brief, clinical eligibility for receipt of ART in BC is based on guidelines generated by the BC-CfE Therapeutic Guidelines Committee, and have remained consistent with current recommendations by the International AIDS Society – USA since 1996 [30]. Currently, the most frequently prescribed initial triple combination ART regimens in BC consist of a nucleoside reverse transcriptase inhibitor (NRTI) and a nucleotide reverse transcriptase inhibitor or two NRTIs as a backbone, plus either i) a non-nucleoside reverse transcriptase inhibitor (NNRTI), or ii) a protease inhibitor boosted with ritonavir (boosted PI) [31].

Prescribing physicians must complete a drug request form, which acts as a legal prescription, to enroll an eligible individual on ART. HIV/AIDS DTP enrollment forms elicit information about patient socio-demographics, HIV-specific drug history, CD4 cell counts, plasma HIV RNA levels, current drug prescription, and the enrolling physician. Prospective clinical, virologic, immunologic and drug-regimen data are collected on a quarterly basis. Epidemiological studies performed on DTP data form the basis of ongoing revisions to the BC-CfE's province-wide HIV treatment guidelines [31]. Patients provide voluntary written informed consent for the BC-CfE to access electronic medical records for research purposes. Ethical approval for these analyses has been provided by the Providence Health Care/University of British Columbia Research Ethics Board.

### Variable Selection

The primary outcome variable of interest was all-cause mortality. This outcome includes individuals who died due to HIV/AIDS-related and non-related causes, including co-morbidities, injuries, accidents, trauma, assaults, drug overdose and suicide. Mortality data was collected on an ongoing basis through physician reports. This data was confirmed through electronic linkage to the British Columbia Division of Vital Statistics registry, which has been shown to capture upwards of 96% of all deaths in the province [32]. Study participants and individuals lost to follow-up were censored on Sept 30, 2011.

#### Primary explanatory variable

The primary explanatory variable of interest was food insecurity, captured cross-sectionally at baseline in 1998/1999 as part of the DTP enrollment form for participants newly initiating ART. Food insecurity was measured using an abbreviated version of the Radimer/Cornell scale. The Radimer/Cornell scale assumes that food insecurity comprises four distinct components that are experienced distinctly at household and individual levels. The scale poses questions regarding food depletion and intake (quantitative component), suitability and adequacy of food (qualitative component), feelings of anxiety and deprivation associated with food (psychological component), and patterns of food acquisition and eating (social component) [33], [34], and has been extensively validated in North America [33], [35]. Since the Radimer/Cornell scale was published in 1990, operational definitions and measurements of food insecurity have continued to evolve [36], [37]. The Radimer/Cornell scale understood ‘hunger’ to be a quantitative component of food insecurity, experienced purely at the individual level [33], [34], and defined as an “*inability to acquire or consume an adequate quality or sufficient quantity of food in socially acceptable ways, or the uncertainty that one will be able to do so*” [36]. A 2006 review of food insecurity and hunger measures by an expert panel for the United Sates Department of Agriculture (USDA) concluded that hunger should be considered an “*indicator and possible consequence of food insecurity*,” and be measured “*distinct from, but in the context of, food insecurity*” [37]. Conceptual research in the field of HIV/AIDS further emphasizes the need for multi-dimensional indicators to capture different dimensions (utilization, access, availability, stability) of food insecurity, and for stand-alone indicators to measure sub-components of food insecurity (quantity, quality, safety). Reconsideration of how food insecurity is measured is required to capture the complex experiences of food insecurity among people living with HIV/AIDS, to generate ‘actionable’ findings for public health programs and policies [38].

The analytic approach to measuring food insecurity and hunger in this study diverged from Radimer/Cornell's and USDA's published methodologies [33], [39]. Participants were categorized as experiencing household food insecurity if they gave a minimum of one positive answer (often/sometimes) to any one of the eight items measuring household food insecurity, which provided a conservative estimate of food insecurity. In the absence of a validated single-question measure of hunger to ascertain food insufficiency at the individual level [37], [38], and in light of public health policy and program requests in our setting to understand what sub-component (quantity, quality, safety) of food insecurity may be driving associations between food insecurity and adverse health outcomes, we extracted and analyzed hunger at the individual level, defined as responding ‘often/sometimes’ to the question: “*I am often hungry, but don't eat because I can't afford enough food*” [33], [34].

#### Secondary explanatory variables

Secondary explanatory variables hypothesized to confound the relationship between food insecurity and mortality were selected based on findings from previous literature, including a previous study examining the impacts of food insecurity on mortality among HIV-positive individuals in this setting [16]. Socio-demographic variables included: age at ART start date (continuous); gender (male *vs.* female); Aboriginal ancestry (yes *vs.* no); annual income (>CAD$15,000 *vs.* ≤CAD$15,000), with a dichotomous split based on Canada Revenue Agency's low income threshold [40]; education (>high school *vs.* ≤high school graduation); and unstable housing (yes *vs.* no), defined as living in a hotel, boarding house, group home, jail, on the street, or having no fixed address at the time of the survey. Clinical variables considered in this analysis included nutritional status, measured by physician and self-reported body mass index (BMI) (kg/m^2^) and calculated using the formula: weight/(height)^2^. Cut-offs for underweight status were based on current WHO standards for HIV-positive individuals, defined as <18.5 kg/m^2^ (underweight) *vs.* ≥18.5 kg/m^2^ (not underweight) [41]. BMI reflects lean body mass and fat mass, has been shown to detect malnutrition at an earlier stage than other anthropometric measures, and is considered a sensitive screening tool for malnutrition among people living with HIV/AIDS [42]. Other clinical variables included use of triple combination highly active antiretroviral therapy (HAART) (yes *vs.* no); PI-based regimen (yes *vs.* no); AIDS diagnosis (yes *vs.* no), defined according to CDC classification [43]; plasma HIV RNA viral load (per log_10_ copies/mL), measured at most recent date prior to survey; CD4 cell counts (per 100 cells/µL), recorded at most recent date prior to survey using flow cytometry and fluorescent monoclonal antibody analysis (Beckman Coulter, Inc., Mississauga, Ontario, Canada); and finally ART adherence, measured on the basis of prescription refill compliance [44], defined as the number of days ART was dispensed over the number of days an individual was eligible for ART in the past 12 months (≥95% *vs*. <95%), at most recent date prior to survey. This adherence variable has shown to reliably predict survival among IDU in previous studies [44], [45]. All potential explanatory variables were collected at baseline in 1998/1999 from the HIV/AIDS DTP enrollment survey, except where indicated above.

### Statistical Analysis

As a first step, bivariate analyses were performed on the entire study sample at baseline, stratified by food insecurity, hunger and all-cause mortality, respectively. Pearson's Chi-Square tests were used to compare categorical variables. In instances where counts were small (five or less), the Fisher's Exact Test was used. Continuous variables were compared using Wilcoxon Rank Sum Test. Next, two Cox proportional hazard confounder models were constructed to determine the association between food insecurity/hunger and all-cause mortality, controlling for potential confounders. A multivariate model was built using an adaptation of methods described by Greenland and colleagues [46], [47]. This manual backward stepwise approach involved first fitting a full model, including all explanatory variables, and noting the value of the coefficient associated with food insecurity/hunger. Reduced models were then constructed, each removing one secondary explanatory variable from the full set. Comparing the value of the coefficient for food insecurity in the full model and each of the reduced models, secondary variables were removed corresponding to the smallest relative change in the coefficient for food insecurity. This iterative process continued until the maximum change of the value for food insecurity from the full model exceeded 5%. The intent of this model building strategy was to retain secondary variables in the final multivariate model with greater relative influence on the relationship between food insecurity and mortality. This technique has been previously applied in studies of HIV-positive individuals to estimate the independent relationship between a hypothesized predictor variable and clinical outcome [48], [49]. As a sub-analysis, this process was repeated to examine the relationship between hunger and mortality. *Ad hoc* tests to assess potential cohort effects on survival were not deemed necessary since participants were all recruited after the introduction of ART, which led to a homogenous trend in reductions of HIV-related mortality over time [50]. All statistical analyses were completed using R v2.10.1 (R Foundation, Vienna, Austria).

## Results

Baseline characteristics of IDU enrolled in the BC-wide HIV/AIDS DTP in years 1998/1999, stratified by food security status, are show in Table 1. A total of 254 individuals enrolled at baseline, responded to the food security scale question in the survey, and had consistent follow-up during the years under study. Of this analytic sample, 181 (71.26%) reported being food insecure, and 108 (42.5%) were hungry; the median age was 38.0 years [interquartile range [IQR]: 34.0–43.0]; 211 (83.07%) were male; 58 (22.9%) reported Aboriginal ancestry; and 219 (96.9%) had a BMI above 18.5 kg/m^2^. The median CD4 cell count was 380.0 per 100 cells/µL (IQR: 220.0–510.0); the median viral load was 2.6 log_10_ copies/mL (IQR: 2.6–3.7); 63 (24.8%) were diagnosed with AIDS; and 123 (48.4%) were ≥95% adherent to ART in the 12 months preceding enrollment. During the study period (between June 21, 1998 and Sept 30, 2011), a total of 105 (41.34%) individuals died. Bivariate comparison of participant characteristics by food security status revealed that individuals with lower incomes, receiving a protease inhibitor (PI)-based regimen, and initiating ART at a later year, were all significantly more likely to be food insecure (*p*<0.05). Median follow-up time was 140.61 months (IQR: 59.63–151.20), and median survival time was 61.04 (IQR: 31.11–83.02). A total of 87 (48.1%) individuals who reported being food insecure died over the study period, compared to 18 (24.7%) individuals who reported being food secure (*p* = 0.001). A total of 54 (51.4%) individuals reporting hunger died, compared to 54 (36.2%) reporting no hunger (*p* = 0.022). Product limit survival estimates revealed that the death rate of the 254 IDU who answered the food security and hunger questions on the baseline survey did not differ from other IDU initiating ART during the same time period.

**Table 1:** Baseline characteristics among HIV-positive injection drug users initiating antiretroviral therapy across British Columbia, by food security status, between June 1998 and Sept 2011 (n = 254).

Characteristic	TotalN (%)	Food Insecure181 (71.3%)	Food Secure73 (28.7%)	*p* - value
**Age**				
Median, IQR^1^	38.0 (34.0–43.0)	38.0 (34.0–43.0)	38.0 (34.0–43.0)	0.933
**Gender**				
Male	211 (83.1%)	148 (81.8%)	63 (86.3%)	0.492
Female	43 (16.9%)	33 18.2%)	10 (13.7%)	
**Aboriginal ancestry**				
Yes	58 (22.9%)	45 (25.0%)	13 (17.8%)	0.286
No	195 (77.1%)	135 (75.0%)	60 (82.2%)	
**Unstable housing**				
Yes	28 (11.8%)	21 (12.2%)	7 (10.8%)	0.936
No	209 (88.2%)	151 (87.8%)	58 (89.2%)	
**Education status**				
≥High school	161 (64.7%)	110 (61.5%)	51 (72.9%)	0.122
<High school	88 (35.3%)	69 (38.5%)	19 (27.1%)	
**Annual income**				
>$15,000	63 (28.1%)	23 (14.7%)	40 (58.8%)	<0.001
≤$15,000	161 (71.9%)	133 (85.3%)	28 (41.2%)	
**Body Mass Index**				
≥18.5 kg/m^2^	219 (96.9%)	153 (96.2%)	66 (98.5%)	0.629
<18.5 kg/m^2^	7 (3.1%)	6 (3.8%)	1 (1.5%)	
**AIDS diagnosis**				
Yes	63 (24.8%)	44 (24.3%)	19 (26.0%)	0.899
No	191 (75.2%)	137 (75.7%)	54 (74.0%)	
**ART start year**				
Median, IQR^1^	1997 (1995–1998)	1997 (1996–1998)	1996 (1994–1998)	0.015
**HAART use^2^**				
Yes	191 (75.2%)	138 (76.2%)	53 (72.6%)	0.655
No	63 (24.8%)	43 (23.8%)	20 (27.4%)	
**PI-based regimen^3^**				
Yes	154 (60.6%)	101 (55.8%)	53 (72.6%)	0.019
No	100 (39.4%)	80 (44.2%)	20 (27.4%)	
**Adherence to ART^4^**				
≥95%	123 (48.4%)	82 (45.3%)	41 (56.2%)	0.153
<95%	131 (51.6%)	99 (54.7%)	32 (43.8%)	
**CD4 cell count** (per 100 cells/µL)				
Median, IQR^1^	380 (220–510)	360 (210–500)	400 (230–555)	0.149
**Plasma HIV RNA** (per log10 copies/mL)				
Median, IQR^1^	2.6 (2.6–3.7)	2.6 (2.6–3.8)	2.6 (2.6–3.2)	0.250
**All-Cause Mortality**				
Yes	105 (41.3%)	87 (48.1%)	18 (24.7%)	0.001
No	149 (58.7%)	94 (51.9%)	55 (75.3%)	

1 Inter-quartile range

2 Highly active antiretroviral therapy use

3 Protease Inhibitor-based regimen

4 Within last 12 months of interview

Unadjusted and adjusted analyses of factors associated with mortality among IDU are presented in Table 2. In unadjusted analyses (Column 1), participants who were food insecure were more than twice as likely to die, compared to individuals who were food secure (hazard ratio [HR] = 2.41, 95% Confidence Interval [CI]: 1.45–4.01). Participants who were hungry were almost twice as likely to die (HR = 1.78, 95% CI: 1.21–2.61) compared to individuals with no hunger. Other factors significantly associated with all-cause mortality included Aboriginal ancestry, low income, <95% adherence to ART, lower median CD4 cell count and higher median plasma HIV RNA. In adjusted analyses, controlling for potential confounders, food insecurity remained significantly associated with all-cause mortality (adjusted hazard ratio [AHR] = 1.95, 95% CI: 1.07–3.53) (Column 2), and hunger was no longer significant (AHR = 1.05, 95% CI: 0.65–1.70) (Column 3). Additional factors associated with increased likelihood of death in the model examining food insecurity included having an annual income >$15,000, Aboriginal ancestry, and a higher median viral load. In the model exploring hunger, additional characteristics associated with higher risk of mortality included older age, annual income >$15,000 and a higher median viral load.

**Table 2:** Unadjusted and adjusted factors associated with all-cause mortality among HIV-positive injection drug users initiating highly active antiretroviral therapy in British Columbia, between June 1998 and Sept 2011 (n = 254).

Characteristic	Unadjusted modelHZ^1^ (95% CI)^2^	Adjusted model including food insecurityAHZ^3^ (95% CI)^2^	Adjusted model including hungerAHZ^3^ (95% CI)^2^
**Food insecure**			
Yes vs. no	2.41 (1.45–4.01)	1.95 (1.07–3.53)	–
**Hunger**			
Yes vs. no	1.78 (1.21–2.61)	–	1.05 (0.65–1.70)
**Age**			
Per 10 year increase	1.19 (0.94–1.50)	1.27 (0.98–1.65)	1.46 (1.09–1.94)
**Gender**			
Male vs. female	0.64 (0.41–1.01)	–	0.59 (0.34–1.02)
**Aboriginal ancestry**			
Yes vs. no	1.92 (1.27–2.92)	2.15 (1.34–3.45)	–
**Unstable housing**			
Yes vs. no	1.50 (0.87–2.60)	–	0.92 (0.46–1.82)
**Education status**			
>High school vs. ≤high school	0.85 (0.57–1.25)	–	–
**Annual income**			
>$15,000 vs. ≤$15,000	0.27 (0.14–0.50)	0.33 (0.16–0.68)	0.28 (0.14–0.58)
**AIDS diagnosis**			
Yes vs. no	0.95 (0.61–1.49)	–	–
**Body Mass Index**			
≥18.5 kg/m^2^ vs. <18.5 kg/m^2^	0.74 (0.27–2.01)	–	–
**ART start year**			
Per year increase	1.03 (0.93–1.14)	0.88 (0.78–1.00)	0.93 (0.81–1.06)
**HAART use^5^**			
Yes vs. no	1.21 (0.76–1.93)	–	–
**PI-based regimen^6^**			
Yes vs. no	0.69 (0.47–1.01)	–	–
**Adherence to ART^7^**			
≥95% vs. <95%	0.59 (0.40–0.87)	–	0.77 (0.48–1.22)
**CD4 cell count**			
Per 100 increase	0.89 (0.82–0.98)	0.96 (0.87–1.06)	0.96 (0.86–1.07)
**HIV RNA viral load**			
Per Log_10_ increase	1.50 (1.23–1.84)	1.42 (1.12–1.80)	1.36 (1.05–1.75)

1 Hazard Ratio

2 95% Confidence Interval

3 Adjusted Hazard Ratio

4 Highly active antiretroviral therapy

5 Protease Inhibitor

6 Within the last 12 months of interview

**Figure 1:**
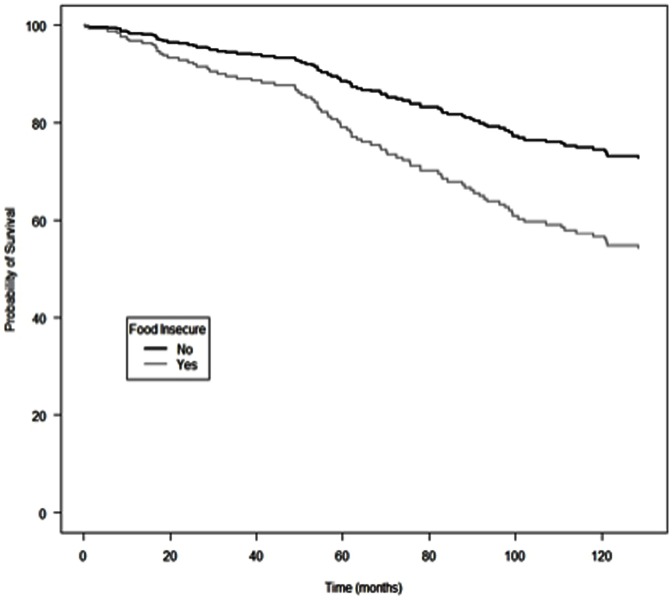
Adjusted cumulative incidence of all-cause mortality among HIV-positive injection drug users initiating antiretroviral therapy in British Columbia, stratified by food security status.

**Figure 2:**
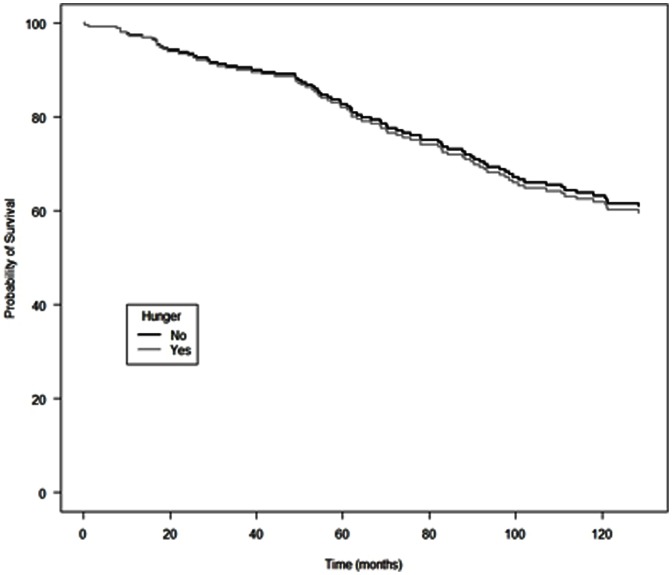
Adjusted cumulative incidence of all-cause mortality among HIV-positive injection drug users initiating antiretroviral therapy in British Columbia, stratified by hunger status.

Adjusted Kaplan-Meir survival probabilities for IDU in this BC-wide cohort, stratified by food insecurity and hunger status, are presented in Figures 1 and 2, respectively. Consistent with findings from the multivariate analyses, individuals reporting food insecurity had significantly reduced probability of survival, compared to individuals who were food secure; and individual reporting hunger did not have lower probability of survival compared to individuals reporting no hunger.

## Discussion

This study builds on our existing body of research regarding the relationship between food insecurity and mortality among people living with HIV/AIDS [16]. This study is the first to examine the potential impact of food insecurity and hunger on mortality among HIV-positive IDU. Mortality rates were elevated in this sample of HIV-positive IDU. After 13.3 years of follow-up, individuals who reported being food insecure at baseline were almost twice as likely to die, when controlling for potential confounders. Hunger was associated with increased risk of death in univariate analysis, but the association was no longer significant after controlling for potential confounders in the adjusted analyses. Our results suggest that addressing food insecurity, in addition to other known social and structural barriers to ART adherence and virologic suppression among illicit drug users [51], [52], such as incarceration [53], homelessness [48], and gender-related factors [54], may be of paramount public health importance.

The finding that food insecurity, and not hunger, was significantly associated with all-cause mortality suggests that other aspects of food insecurity, measured within the Radimer/Cornell scale, may be driving this association, including poor dietary diversity and/or anxiety regarding food access. Food insecurity is theorized to be linked to adverse HIV-related outcomes through diverse nutritional, mental health and behavioral pathways [25]. Macro- and micronutrient deficiencies, which represent an extreme consequence of, and surrogate markers for, poor dietary diversity, have been associated with mortality among HIV-positive individuals in the ART era. Compared to non-IDU, active IDU may be at increased risk of developing nutrient deficiencies due to metabolic abnormalities and nutrient interactions associated with drug use [55]. Low albumin and phosphate levels have been associated with early mortality among malnourished individuals initiating ART [7], [9]. Deficiencies in zinc, Vitamin A, iron and B_12_ have also been associated with increased risk of HIV-related mortality in illicit drug-using populations [5], [13], [55], [56]. In particular, selenium deficiency is associated with a 20-fold increase in risk of mortality among HIV-positive IDU [55]. Findings from the current study, taken together with existing evidence regarding the relationship between nutrient deficiencies and mortality among HIV-positive IDU populations, suggest an urgent need for public health bodies to evaluate the possible role of screening for malnutrition and food supplementation to prevent excess mortality among IDU receiving ART in this setting.

The observed association between food insecurity and all-cause mortality in this sample of IDU receiving ART may also be explained by mental health mechanisms. Two questions on the Radimer/Cornell scale pertain specifically to feelings of anxiety regarding food access: “I *worry whether my food will run out before I get money to buy more” and “I worry about whether the food that I can afford to buy for my household will be enough.*” Obsessive or chronic worrying is recognized as a symptom of generalized anxiety disorder within the DSM-V [57]. Mental health disorders, including anxiety and depression, are commonly reported among HIV-positive populations, and believed to increase risk of mortality through both behavioral and biologic mechanisms. Symptoms of depression have been associated with poor virologic response [58], [59], [60], reduced immunologic capacity [61], and AIDS and non-AIDS related death among individuals on ART [49], [62], [63], [64], [65]. Feelings of guilt, fear and discrimination have been associated with delayed access to HIV treatment and care [66], and non-adherence to ART [67], [68], [69]. A recent study of 9,003 HIV-positive individuals in the US found that presence of mental health disorders, including schizophrenia and bi-polar disorder, were significantly associated with all-cause mortality [70]. Taken together with this previous evidence, findings from this study suggest a need to evaluate the possible role of comprehensive mental health support in the context of existing harm reduction and HIV treatment services in order to prevent excess mortality.

This study has several strengths and limitations that warrant consideration. Participants were not randomly selected, and therefore are not representative of the general HIV-positive or IDU populations in BC. Common to all survival analyses, the censoring of participants who were either event-free or lost to follow-up may have led to an underestimation of true time to event. Longitudinal data was not available for most socio-demographic and clinical variables. A major limitation of the study is that food security and hunger status were only collected at baseline. This study was therefore unable to ascertain the possible time-updated effects of food insecurity/hunger on mortality. Multivariate models in this study controlled for several variables that have been hypothesized to be on the causal pathway between food insecurity and mortality [25], and may have therefore underestimated true effect size. Information bias, and specifically responder bias, may have led to non-differential misclassification of hunger status, biasing Odds Ratio estimates towards the null. Residual confounding – due to dichotomization of continuous variables, use of surrogate markers, misclassification, or failure to account for unobserved/unknown confounders – may have introduced bias into effect estimates. Future studies could be strengthened by considering the potential confounding impact of geographic region on the relationship between hunger and mortality, which has been independently associated with both HIV-related food insecurity (data not published) and mortality trends in BC [50].

Although the Radimer/Cornell scale is not considered the most contemporary of food insecurity measurement options, select measures within the scale have been incorporated into contemporary food security scales, including the United States Household Food Security Survey Module (HFSSM) [71] and Canadian versions of the module [72], offering some degree of geographic and temporal comparability. Notably, the hunger measure used in our analysis has remained consistent across different food security modules developed since the early 1990s, with the only difference being an emphasis on hunger frequency and duration in contemporary tools [37]. The advantage of the Radimer/Cornell scale used in this study is that it prompted respondents for ‘current’ food insecurity and hunger status, minimizing recall bias.

Because survey data were self-reported, this study may have also been susceptible to recall bias and social desirability bias. While the measure of hunger used in this study has been extensively validated in low income populations [27], [73], [74], future studies could be strengthened by applying robust dietary intake assessment methods validated for use among HIV-positive individuals, including 24 hour dietary recall and food frequency questionnaires [75], [76]. Use of self-reported weight and height for BMI may have been prone to bias, with studies demonstrating that individuals tend to under-report energy intake, weight and BMI, and over-report height [77]. Future studies could additionally consider clinical assessment of body weight loss and body cell mass, and subjective assessments of global nutritional status, which have found to be robust among people living with HIV/AIDS [42], and body composition and biochemical measures commonly used in HIV-positive IDU populations [76].

In summary, we found that food insecurity was associated with a two-fold increased risk of mortality, after controlling for potential confounders, among HIV-positive IDU receiving ART across BC, Canada. Further research is necessary to understand the mechanisms through which food insecurity drives this association, and to examine modifying effects of diverse nutritional, mental health and behavioral factors in the relationship between food insecurity and mortality. Public health organizations should prospectively evaluate the possible role of food supplementation and socio-structural supports on survival among IDU within HIV treatment programs.
